# The psychophysiology of guilt in healthy adults

**DOI:** 10.3758/s13415-023-01079-3

**Published:** 2023-03-25

**Authors:** Chloe A. Stewart, Derek G.V. Mitchell, Penny A. MacDonald, Stephen H. Pasternak, Paul F. Tremblay, Elizabeth Finger

**Affiliations:** 1 Graduate Program in Neuroscience, Schulich School of Medicine and Dentistry, University of Western Ontario, 1151 Richmond St, London, ON N6A 3K7 Canada; 2 Department of Psychiatry, Schulich School of Medicine and Dentistry, University of Western Ontario, London, ON Canada; 3 Department of Psychology, University of Western Ontario, London, ON Canada; 4 Department of Anatomy and Cell Biology, University of Western Ontario, London, ON Canada; 5 The Brain and Mind Institute, University of Western Ontario, Ontario, London, ON Canada; 6 Department of Clinical Neurological Sciences, University of Western Ontario, London, ON Canada; 7 Robarts Research Institute, Schulich School of Medicine and Dentistry, University of Western Ontario, London, ON Canada; 8 Parkwood Institute Research Program, Lawson Health Research Institute, London, ON Canada

**Keywords:** Guilt, Psychophysiology, Social emotions

## Abstract

Guilt is a negative emotion, elicited by realizing one has caused actual or perceived harm to another person. Anecdotally, guilt often is described as a visceral and physical experience. However, while the way that the body responds to and contributes to emotions is well known in basic emotions, little is known about the characteristics of guilt as generated by the autonomic nervous system. This study investigated the physiologic signature associated with guilt in adults with no history of psychological or autonomic disorder. Healthy adults completed a novel task, including an initial questionnaire about their habits and attitudes, followed by videos designed to elicit guilt, as well as the comparison emotions of amusement, disgust, sadness, pride, and neutral. During the video task, participants’ swallowing rate, electrodermal activity, heart rate, respiration rate, and gastric activity rate were continuously recorded. Guilt was associated with alterations in gastric rhythms, electrodermal activity, and swallowing rate relative to some or all the comparison emotions. These findings suggest that there is a mixed pattern of sympathetic and parasympathetic activation during the experience of guilt. These results highlight potential therapeutic targets for modulation of guilt in neurologic and psychiatric disorders with deficient or elevated levels of guilt, such as frontotemporal dementia, posttraumatic stress disorder, and Obsessive-compulsive disorder.

**Supplementary Information:**

The online version contains supplementary material available at 10.3758/s13415-023-01079-3.

Guilt, a negative, moral emotion, has powerful functions to encourage prosocial behaviour and to limit antisocial behaviour. Guilt is caused by the awareness that one has performed, might perform, has been, or could be the beneficiary of an action or inaction that has caused or could cause harm or inequality to befall another party (Huhmann & Brotherton, 1997; Zeelenberg & Breugelmans, 2008). In popular culture and in anecdotal experiences, guilt often is described as a visceral, embodied emotion, the physical components of which are intrinsically linked to its emotional experience (Boden & Eatough, 2019; Day & Bobocel, 2013; Norbury, 2012; Tangney et al., 1996). However, little is known concretely about the physiologic components that attend guilt beyond the anecdotal. While it is well established that basic emotions, such as happiness and fear, generate patterns of autonomic activation in the body (Kreibig, 2010; Pace-Schott et al., 2019), whether guilt produces a detectable autonomic output in healthy adults has not yet been comprehensively examined.

## Guilt, shame, and embarrassment

Two related social emotions often overlap and co-occur with guilt. These are shame—a negative self-evaluation in the face of a behaviour, thought, or feeling that is fundamentally incongruent with one’s self-concept—and embarrassment—awareness of loss of esteem or face due to having been witnessed committing some error, accident, or faux pas (Niedenthal et al., 1994; Tangney et al., 1996). Embarrassment is distinguished from guilt in three ways. Embarrassment absolutely requires an audience or observer, real or imaginary, whereas guilt requires none (Withers & Sherblom, 2008). Embarrassment is typically felt as more transitory and less intense than guilt and tends not to evoke additional negative emotions, such as sadness or anger (Miller & Tangney, 1994; Tangney et al., 1996). In embarrassment, the individual takes less responsibility for the act that triggered the feeling than they would in guilt (Miller & Tangney, 1994; Keltner and Buswell, 1997). Shame is similarly distinguished from guilt in three ways. In shame, the attribution for blame is placed on the self, not on the behaviour; the opposite is true in guilt (Miller & Tangney, 1994; Niedenthal et al., 1994). Shame is experienced as more psychologically aversive and a more intense emotion than guilt (Tangney et al., 1996). When experienced, shame drives individuals to withdraw, to deny, and to lash out at others, whereas guilt drives to repair and ameliorate the situation (Drummond et al., 2017; Pivetti et al., 2016; Tangney et al., 2011).

## Autonomic nervous system and emotion

The autonomic nervous system (ANS) coordinates the unconscious regulation of the body via its two major subdivisions: the sympathetic nervous system (SNS), responsible for activation of the body in response to external or internal threat, and the parasympathetic nervous system (PSNS), responsible for energy conservation (Jänig, 2006; Jänig & Häbler, 2003; McCorry, 2007). Studies have demonstrated that the ANS is activated by and during the experience of basic emotions, such as anger, fear, and happiness (Kreibig, 2010; Pace-Schott et al., 2019). While both divisions are tonically active at all times, and the experience of each emotion often involves mixed SNS and PSNS activation and withdrawal, there are some clear patterns of activation. The SNS is activated principally in emotions which require immediate behavioural preparedness and orientation towards the stimulus, such as fear, anxiety, or anger (Aue et al., 2007; Baldaro et al., 1996; Christie & Friedman, 2004; Stemmler et al., 2007). The PSNS, by contrast, predominates in emotions that involve soothing or relaxing, such as relief or contentment (Bradley et al., 2008; Chan & Lovibond, 1996; Christie & Friedman, 2004; Palomba et al., 2000), or emotions that involve passivity or a lack of available behaviours to respond to or ameliorate them, such as sadness (Britton et al., 2006; Christie & Friedman, 2004; Gross et al., 1994; Kreibig, 2010).

Best practices for study of emotion and the ANS include use of a within-subject design to help mitigate individual variability and reduced impact of external factors and context effects on physiologic parameters (Quintana & Heathers, 2014; Stemmler, 2003). In research eliciting emotions, comparison across several emotions and use of a stimulus or task close to the stimulus or task of interest as a comparison is critical to reduce effects driven by attention and task demands. (Levenson, 2003; Quintana & Heathers, 2014). Given known interactions between different facets of the ANS, and the complexity of ANS responses in emotion, the use of single or limited psychophysiologic measures has been a noted limitation of much of the extant literature (Kreibig, 2010; Stemmler, 2003). Inclusion of multiple physiologic indicators of potential emotional reactivity, including cardiac, respiratory, electrodermal, mesenteric, and swallowing related metrics, modeling both linear and nonlinear effects (Quintana & Heathers, 2014) is recommended to elucidate the integrated and complex response of the ANS to emotion elicitation.

## Autonomic nervous system and guilt

Despite the anecdotal descriptions of bodily sensations associated with guilt, little is known about the physiologic components that attend guilt. Some researchers have investigated the ANS in relation to other closely related social emotions, particularly embarrassment. In both firsthand and vicarious embarrassment, the SNS has been observed to predominate as indexed primarily by both cardiovascular and electrodermal indices, and occasionally by skin temperature or facial plethysmography (Gerlach et al., 2003; Harris, 2001; Hofmann et al., 2006; Miller, 1987; Miller & Fahey, 1991; Müller-Pinzler et al., 2012; Shearn et al., 1990). Shame has similarly been associated with predominance of the SNS, as measured by heart rate (Herrald & Tomaka, 2002).

Indirect support of involvement of the ANS in guilt also comes from studies of individuals with maladaptive guilt. In anxiety disorders where guilt is excessive, such as posttraumatic stress disorder (PTSD), obsessive-compulsive disorder (OCD), or generalized anxiety disorder, hyperreactivity of, and hypervigilance to, the ANS has been observed (De Zorzi et al., 2021; Domschke et al., 2010; Hoehn-Saric & McLeod, 2000; Murphy et al., 2017; Pruneti et al., 2010, 2016). By contrast, in disorders where guilt is deficient, such as psychopathy, underreactivity or absence of ANS response to emotion, and low awareness of the ANS in general, has been observed (Fung et al., 2005; Ishikawa et al., 2001; Lyons & Hughes, 2015; Nentjes et al., 2013).

In typically developing children, guilt-inducing transgressions have been linked with activation of the SNS based on peripheral nasal vasoconstriction, and with PSNS activity via respiratory sinus arrhythmia withdrawal and heart rate deceleration (Colasante et al., 2018; Ioannou et al., 2013; Malti et al., 2016). Further studies have linked inappropriately high or low PSNS activation—measured through resting heart rate, respiratory sinus arrhythmia, and skin conductance—with low guilt and high transgressiveness in children (Colasante et al., 2020; Colasante & Malti, 2017). Taken together, these findings suggest that in healthy children there is ANS reactivity to guilty feelings and that SNS and PSNS co-dominate in this experience. How and the extent to which the various components of the ANS are engaged in the experience of guilt has never been established in healthy adults.

## Present study

Because the extant literature suggests a relationship between guilt and the ANS, but has not yet characterized this relationship, the objective of the present study was to investigate and identify autonomic signals important for distinguishing guilt from other emotions in healthy adults. We hypothesized that the experience of guilt would be associated with a pattern of autonomic activations common across individuals and distinct from other emotions. Based on the existing literature surrounding the bodily experience of related emotions, particularly embarrassment and shame, as well as the behaviour-focused, motivational drive of guilt, we predicted that the overall ANS pattern would feature greater relative activation of the SNS during the experience of guilt compared to other emotions (Gerlach et al., 2003; Jansen et al., 1995). Healthy adults were recruited to participate in a video task designed to elicit guilt during the continuous monitoring of psychophysiological signals.

## Method

### Transparency and openness

We report how we determined our sample size (below), all data exclusions, all manipulations, and all measures in the study, and we follow JARS (Kazak, 2018). All data are available at 10.17605/OSF.IO/AVD37. Data were analyzed by using R Studio v1.3.959 (R Core Team, 2018; RStudio Team, 2016). See Analytic Approach for further details. This study’s design and its analysis were not preregistered.

## Participant characteristics and enrollment

Healthy adults were recruited in London, Ontario, Canada, between late 2017 and early 2020 through word of mouth or flyers and advertisements inviting interested participants to take part in research on emotion posted on city buses, in community locations, such as libraries and grocery stores, and at Western University. Inclusion criteria were: age 18 to 80 years; normal or corrected-to-normal vision; normal or corrected-to-normal hearing; and fluency in English. Exclusion criteria were any current major neurological or psychological disorder or the use of beta blockers. All study procedures were approved by the University of Western Ontario Research Ethics Board. Participants provided written, informed consent before undertaking study procedures and were compensated for their time.

### Sample size calculations

A sample size of 100 was targeted to maintain a minimum power (1-ß) of 0.95 and detect a small effect size between 0.14 and 0.23 with alpha = 0.05. Power calculations were performed by using G* Power 3.1.7 (Faul et al., 2007) based on a MANOVA procedure with one group and six response variables. The calculation was based on estimates from a study of embarrassment psychophysiology, which detected significant group effects with similar measures and tasks (Müller-Pinzler et al., 2012). A final sample size of 95 was achieved.

## Stimuli

### Opinions and behaviour questionnaire

Participants were asked to complete a computer-based 103-item questionnaire, which they were informed would extract their opinions and behaviours on several topics, including charitable giving, environmental conservation, and national identity (see supplement). This questionnaire was developed by the authors based on questionnaires on similar topics created by Statistics Canada (Statistics Canada, 2021). Participants responded by using yes/no, a scale from 1 (*not at all*) to 5 (*very much*), multiple choice, or free answer depending on the question. Before beginning the questionnaire, participants were informed that their responses would generate feedback about themselves that they would receive during the video task and that this feedback would be based on previous survey responses (see below).

### Feedback statements

After completing the questionnaire and before undertaking the video task, participants were reminded that they would see feedback statements that would provide true feedback about themselves, allegedly based on comparisons to Statistics Canada and previous participants. Before the onset of every video clip a linked short statement purporting to be derived from the opinions and behaviour questionnaire was presented (see supplement). Each video had only one statement associated with it, which directly related to the video content. Thus, regardless of their responses on the questionnaire, every participant received the same standard set of feedback statements. These statements were designed to make each video clip personally relevant to the participant, specifically by beginning with “You…” and containing either a comparison of themselves to others or a description of themselves or their behaviour (i.e., “You bake less than the average Canadian” or “You feel connected to Canada”) that was related to the video’s content to maintain consistency between the comparison emotions and the guilt condition. For the guilt condition, feedback statements were written to enhance the experience of guilt by informing the participant that the behaviours they reported in the questionnaire were harmful or that their inaction was bringing harm. For example, before a video about starving children in need of donations, a participant would see “You donate less than the average Canadian,” whereas a video describing the negative environmental impacts of laundry would be preceded by the statement: “Your laundry habits waste more water than two-thirds of Canadians.” All guilt feedback statements were written with the subject put in comparison with an average other, under the assumption that most participants would not know the true engagement of others in civic or charitable behaviours, or else were written as statements that the average person could accept as true about themselves, such as “You sometimes ignore charity appeals.”

### Video clips

Forty short video clips from various television shows, movies, charitable agencies, and advertising campaigns were chosen to elicit the target emotions of guilt, amusement, disgust, neutral, pride, and sadness (see supplement). These emotions were selected to ensure comparisons to emotions closely related to guilt (disgust, sadness), social emotions (pride), an emotion distinct from guilt (amusement), and a baseline nonemotional state (neutral). Ten videos were selected to elicit guilt, whereas six videos were chosen to elicit each of the comparison emotions. These clips were selected by the authors and tested in a pilot study of 14 people (8 females) to ensure that they reliably elicited the target emotions and to ensure that intensity, arousal, and valence ratings were consistent across emotion categories. Clips lasted from 20 seconds to 2 minutes, with an average length of 1 minute. The time window in which the emotions occurred most strongly in each video were identified in the pilot study using CARMA video rating software, which enables continuous affective ratings similar to an affective rating dial (Ruef & Levenson, 2007), and only these windows were used in analysis (Girard, 2014).

### State and trait measures

Four self-administered paper questionnaires were chosen to assess the state and trait qualities of the sample. The State-Trait Anxiety Inventory and Body Perception Questionnaire were added to the protocol after the first 36 participants (28 females) had participated in the study.

- ***The Guilt Inventory***—a 45-item questionnaire, was used as a measure of guilt proneness (Jones et al., 2000). Participants rated their level of agreement from 1 (*agree strongly*) to 5 (*disagree strongly*) with a series of statements that are designed to establish their state guilt, trait guilt, and attachment to moral standards and rules.
- ***The Empathy Quotient*** (EQ)—a 60-item questionnaire, assessed trait empathy (Baron-Cohen & Wheelwright, 2004). Participants rated their agreement from 1 (*strongly agree*) to 4 (*strongly disagree*) on questions designed to establish their understanding of and connection to the emotions and opinions of others.
- ***The State-Trait Anxiety Inventory*** (STAI)—a 40-item questionnaire was used as a measure of participant anxiety during the time of testing and in their daily lives (Spielberger et al., 1970). Participants rated their agreement from 1 (*not at all*) to 4 (*very much so*) on a series of statements describing their current level of anxiety, and between 1 (*almost never*) to 4 (*almost always*) on a series of statements describing their usual level of anxiety.
- ***The Body Perception Questionnaire-Short Form*** (BPQ)—a 46-item questionnaire was used to assess awareness of bodily states and autonomic reactivity with three subscales: Body Awareness (sensitivity to internal body feelings and functions), supradiaphragmatic reactivity (responsivity of organs above the diaphragm), and subdiaphragmatic reactivity (responsivity of organs below the diaphragm) (Cabrera et al., 2018). Participants rated from 1 (*never*) to 5 (*always*) their level of awareness of their body and how it typically behaved.

## Procedure

Following informed consent and demographic information collection, participants were placed in the psychophysiological monitors to allow them to become comfortable and familiar with the equipment before testing began. Participants were seated in a comfortable chair in front of a computer monitor and asked to complete the opinions and behaviour questionnaire themselves. Following completion of this task the psychophysiological equipment was turned on and participants received the full task instructions. During data collection, a research coordinator was separated from the participant by a standing screen. This allowed the coordinator to respond to questions, concerns, or emotional distress, while reducing distraction for the participant.

### Video task

The task was programmed and run in E-Prime version 3.0 (Psychology Software Tools, Pittsburgh, PA). A single feedback statement appeared on the screen and remained until the participant clicked to acknowledge it. Participants then viewed the linked video clip. After the video ended, participants were shown a black screen lasting 10 seconds, during which they were instructed to think about the video and what the contents of the video made them feel. Participants then reported via selection from a list of 12 emotion words the primary emotion they felt while watching the clip; participants were allowed to select only one word and instructed to pick the emotion that they felt most strongly during the video. This emotion words list contained the six target emotions as well as words potentially related to guilt (anger, contempt, embarrassment, shame), and remaining basic emotions (fear, happiness).

**Fig. 1:**
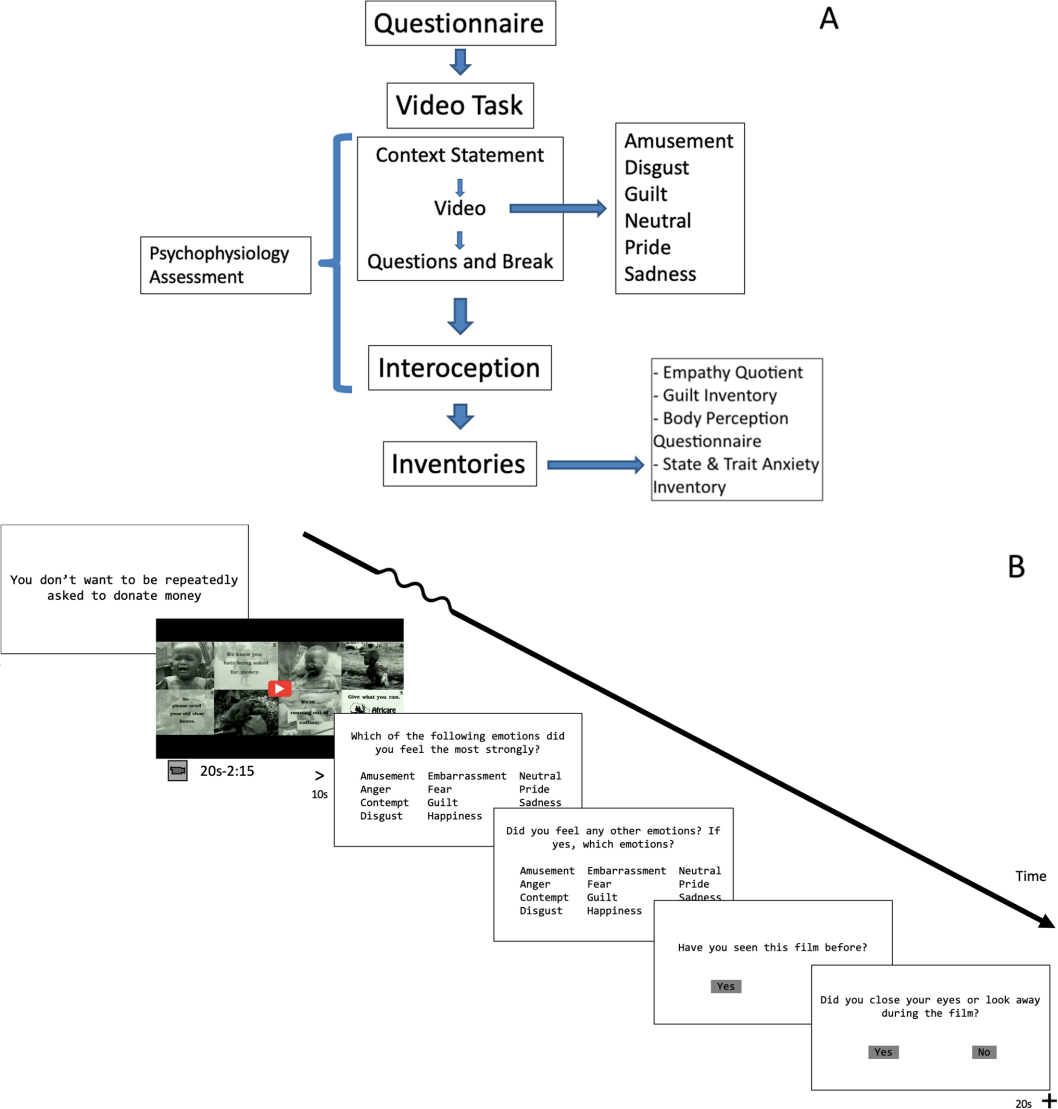
Overall study design **(A).** Schematic of the video trial design, depicting context statement, emotional video, and post-video questions (**B**)

Participants were then presented with the same 12 emotion words and asked to select any additional emotions that they felt while watching the video; during this selection, they were free to pick as many of the words as they felt described their experience, or none. This was followed by a 20-second white screen, which marked a rest period. This repeated in an individually randomized order until the participant had watched all 40 videos (Fig. 1). On average, the entire video task took approximately one hour and twenty minutes.

### State and trait measures

Self-administered paper questionnaires were completed by the participants to characterize the state and trait qualities of the sample (see State and Trait Measures, above).

### Debrief

Following the conclusion of all task activities, a deception check was performed. Participants were asked to rate on a scale from 1 (*agree strongly*) to 5 (*disagree strongly*) whether they believed, on average, that the feedback statements they received were accurate and applied to them. Participants were then debriefed about the nature of the study’s deception and given the opportunity to withdraw their consent to be included in the final analysis.

## Psychophysiological assessment

Psychophysiological data was collected during a baseline 3-minute rest period and for the entirety of the video task. Psychophysiological data was recorded using a Biopac MP160 system at 1 kHz (Biopac Systems Inc., Goleta, CA). All psychophysiological data were collected, cleaned, and analyzed in Biopac’s AcqKnowledge 5.0 software. Electrocardiogram (ECG) signals were recorded using a standard three-electrode system, with an Ag-AgCl electrode placed below the right shoulder, one below the left shoulder, and one near the bottom of the rib cage on the left. Electrodermal activity (EDA) was recorded using two Ag-AgCl electrodes placed on the volar surface of the medial phalanges of the index and middle finger of the participant’s nondominant hand. Swallowing electromyography (EMG) was recorded using a three-electrode configuration with two Ag-AgCl electrodes placed on the right side of the larynx and a ground electrode placed on the right shoulder. Electrogastrography (EGG) was recorded using a standard three-electrode system, with one Ag-AgCl electrode placed an inch above the umbilicus, a second approximately 6 inches away on a 45-degree angle from the first, and a third ground electrode placed above the right hip. Respiration was recorded using the TSD201 Respiratory Effort Transducer, an elasticized belt which was fastened snugly around the participant’s torso at the approximate height of the sternum.

## Psychophysiological data cleaning and analysis

All psychophysiological data was scored within analysis windows delineated by the onset of the emotion as identified in the pilot study and the offset of each video. Because the EGG has a slow response time, the 30 seconds immediately post video offset also was included in the analysis window for this signal. All data were examined for movement artifacts, which were confirmed using discreetly recorded videos of participants taken throughout the task. Movement artifacts were removed from the data once identified. Videos were categorized for analysis based on an individual’s reported emotional experience rather than intended emotion. Videos for which nontarget emotions were identified as the primary emotion, such as shame or embarrassment, were not included in analysis. Data for individual videos were averaged across all videos of the same emotion as identified by the participants to create a composite score for each psychophysiological measure in each emotion. Missing data points for individuals who were missing single data points due to brief technical glitches or failures but for whom the rest of the data was usable were imputed using multivariate imputation by chained equations via the *mice* package version 3.11.0 (Van Buuren & Groothuis-Oudshoorn, 2011) in R Studio v1.3.959 (R Core Team, 2018; RStudio Team, 2016). Imputations had to be performed for .605% of total signals. All psychophysiologic measures were transformed into percent of maximum possible (POMP) scores to account for individual variation and enable comparison between participants (Cohen et al., 1999). Psychophysiologic measures were chosen to reflect standard measurements reported in previous studies of emotion psychophysiology (Cacioppo et al., 2016; Kreibig, 2010).

**Respiratory Sinus Arrhythmia** (RSA) was chosen as a marker of heart rate variability, as it has been well validated as a measure of PSNS control (Berntson et al., 1997; Berntson et al., 2016). RSA data were cleaned and scored using AcqKnowledge’s automated RSA analysis software, which measures the minimum and maximum R-R intervals during a respiration cycle.

**Interbeat Interval** (IBI) was selected as a measure of heart rate that would accurately reflect changes in autonomic branch activation regardless of baseline IBI and which is more sensitive to moment-to-moment changes in short term emotional state (Berntson et al., 1995; Berntson et al., 2007; Lohani et al., 2018). IBI data were calculated by using AcqKnowledge’s automated Find Rate function. Raw heart rate data was converted to IBI by dividing 60,000 by the identified heart rate (Berntson et al., 2016).

**Tonic EDA magnitude** was selected as a measure of electrodermal activity to account for participants who did not display measurable specific skin conductance responses related to the video stimuli (Boucsein, 2012; Dawson, Schell, and Filion, 2016). EDA data was processed through a low pass filter (0.1 Hz). Specific skin conductance responses were identified by using AcqKnowledge’s automated skin conductance response program, which identified any fluctuation of 0.05 microsiemens or greater.

**Respiratory rate** was selected as a simple and effective marker of respiratory effort that has been previously validated (Lorig, 2016). Respiration data were rescaled and processed through a bandpass filter (0.05-1 Hz). Respiration rate was calculated using AcqKnowledge’s automated respiration rate program, which calculates the number of peak-to-peak breath cycles within an identified time window.

**Swallowing rate** was selected as a measurement which has been previously validated in studies of swallowing and emotion (Cuevas et al., 1995; Ritz & Thöns, 2006). Swallowing EMG data were cleaned through the removal of movement, breath and speech artifacts. EMG responses were counted as the number of absolute pulses detected in each analysis window.

**Dominant frequency of the EGG signal** was selected to identify the dominant power spectra in relation to emotional experience (Levine, 2017; Shenhav & Mendes, 2014; Stern, 2002). EGG data was amplified and filtered offline using a bandpass filter (0.01-0.5 Hz) and cleaned of breath contamination by an adaptive filter set to use the respiration channel as noise. The average frequency of each analysis window was extracted using a fast Fourier transform.

## Analytic approach

All data analysis was carried out in R Studio v1.3.959 (R Core Team, 2018; RStudio Team, 2016). To account for nonnormally distributed data and small sample sizes in the state and trait data, tests were performed with bootstrapping where appropriate. Bootstrapped independent samples *t*-tests were carried out using the *boot.ttest2* function in the *Rfast* package v2.0.1 (Papadakis et al., 2018). Bivariate correlations between state and trait variables were performed using the *cor.test* function in the *stats* package v4.1.0 and the *boot* function in the *boot* package v1.3-25 (Canty & Ripley, 2012; Venables & Ripley, 2002). Bootstrapped ANOVAs were performed by using the *aov* function in the *stats* package v4.1.0 and the *boot* function in the *boot* package v1.3-25 (Canty & Ripley, 2012; Venables & Ripley, 2002). Confidence intervals were calculated by using the *confint* function in the *stats* package v4.1.0 (Venables & Ripley, 2002). All graphs were made using the *ggplot2* package v3.3.5, and in-graph calculations were performed using the *ggpubr* package v0.4.0 (Kassambara, 2020; Wickham, 2016).

## Relationship between emotions and psychophysiological signals

To identify the differences in the six psychophysiological measures across the six emotional categories, psychophysiological data were entered into a repeated-measures multivariate analysis of variance (MANOVA), with age entered as a covariate and gender entered as a between-subjects factor. As the psychophysiological data was non-normally distributed, a semiparametric MANOVA, which allows for resampling, was run using the *MANOVA.RM* package v0.4.2 (Friedrich et al., 2021). Using the repeated measures design, each of the six physiological measures was compared within subjects across each of the six emotional experiences to describe the overall pattern of psychophysiological differences between each emotion. To delineate significant effects observed in the MANOVA, multinomial logistic regression was performed, with all psychophysiological signals entered as independent variables and guilt as the reference group. Multinomial logistic regression was performed by using the *multinom* function in the *nnet* package v7.3-16 (Ripley et al., 2016). All reported *p* values are Holm-Bonferroni corrected unless otherwise indicated.

## Trait and state measures

Bootstrapped independent samples *t*-tests were performed to compare means between genders for all state and trait measurements, because previous research has consistently found gender differences on the EQ (Wakabayashi et al., 2006). Bootstrapped bivariate correlations investigated the relationships between the Guilt Inventory, the EQ, the BPQ, the STAI, and selection of guilt as the primary emotion in the video task.

## Results

### Participant demographics

A total of 108 participants (55 females) ranging in age from 18 to 77 years (M = 39, Med = 31) participated in the study. Participants reported attending between 6 and 23 years of formal education (M = 15.963, Med = 16). Participants were excluded from the main analysis for failure to endorse feeling guilt as the primary emotion for any video during the video task (n = 7), technical errors in recording of physiological data (n = 3), and incomplete recording due to power or equipment failure (n = 3). Thus, 95 participants (49 females) were included in the final psychophysiological data analysis.

### Trait and state ratings

Mean values on the EQ (Ohtsubo et al., 2019; Preti et al., 2011), Guilt Inventory (Jones et al., 2000; O’Connor et al., 1999), STAI (Knight et al., 1983), and BPQ (Cabrera et al., 2018) were comparable to previous studies in similar populations (Supplementary Material).

### Task debrief and deception check

No participations requested removal of their data after the debrief. The mode and median response to the deception check question of whether the participants believed the feedback statements given to them were accurate and applied to them was 2 or “Agree somewhat.”

### Psychophysiology results

Psychophysiologic composite scores were created based on reported emotional experience rather than intended emotion. Mean, range, standard deviation, and target accuracy of identified emotion are reported in Table 1. Pride and guilt were most likely to be misidentified, whereas sadness and disgust were most likely to be accurately identified (Table 1).

**Table 1:** Mean, range, and standard deviation for the number of emotional videos included in composite psychophysiological scores, and frequency at which the target emotion of video was endorsed as the primary emotion experienced

Emotion	M	Range	SD	Frequency of primary endorsement of target
Guilt	3.98	1-9	2.00	24%
Amusement	8.50	2-16	3.06	66%
Disgust	4.88	0-8	1.51	79%
Neutral	8.97	1-20	4.16	77%
Pride	3.79	0-11	1.91	56%
Sadness	7.91	2-15	2.57	83%

Given the duration of the task and possibility of reduced attention that may have affected psychophysiologic responding, we analyzed the concordance between the target emotion and emotion endorsed during the first (M = 11.905, SD = 2.733) and second half (M = 12.126, SD = 3.019) of the study and did not find that they significantly differed, *t*(94) = −0.769, *p* = 0.444. We also examined the raw EDA signal over the course of the task, because it would be most likely to change over time. We found no significant differences between the first (M = 2.996, SD = 2.729) and second half (M = 3.028, SD = 2.875) of the protocol, *t*(95) = −0.455, *p* = 0.650, nor between the first (M = 3.032, SD = 2.720) and final quarters (M = 3.078, SD = 2.901), *t*(95) = −0.443, *p* = 0.659.

#### Omnibus MANOVA

**Fig. 2:**
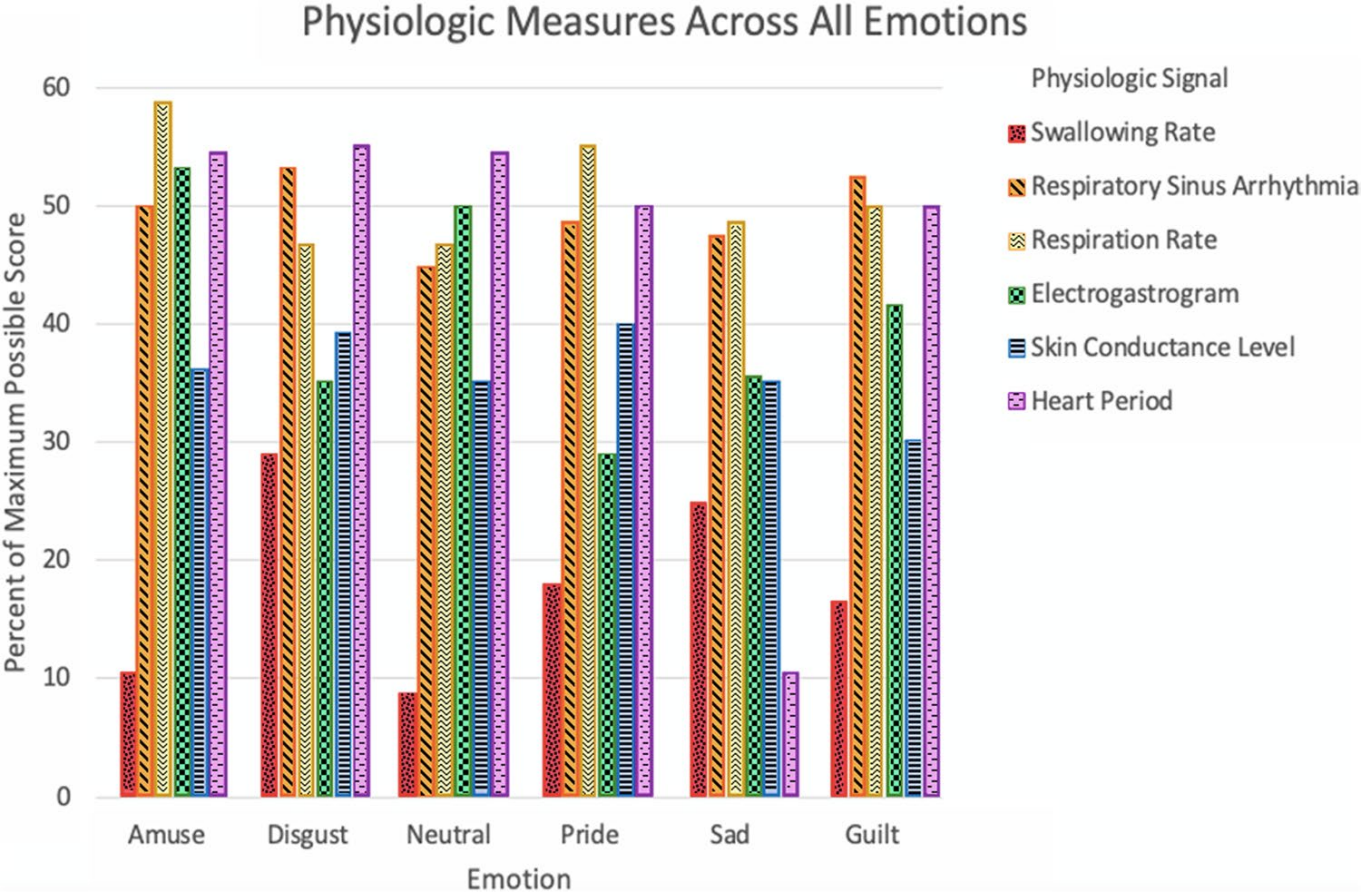
Patterns of psychophysiological measures across all emotions. There was a significant interaction between emotion and physiological signal, F(25, 68) = 3.651, *p* < 0.001, η_p_^2^ = 0.573

Using Pillai’s Trace, there was a significant effect of emotion category on psychophysiological signals, *F*(25, 68) = 3.651, *p* < 0.001, η_p_^2^ = 0.573 (Fig. 2).

#### Multinomial logistic regression

A multinomial logistic regression confirmed that the psychophysiological signals contributed to the prediction of guilt (Table 2). The regression was statistically significant, χ^2^(5) = 12.115, *p* = 0.03. Table 3.

**Table 2:** Results of multinomial logistic regression using guilt as the reference group

	*Amusement*					
	Coeff	SE	z	*p*	95% CI	Odds ratio
Swallowing	−0.020	0.011	−1.817	0.069	−0.04 – 0.002	0.980
RSA	−0.005	0.008	−0.552	0.581	−0.02 – 0.01	0.995
Respiration	0.036	0.010	3.605	<0.001***	0.02 – 0.06	1.037
EGG	0.057	0.012	4.814	<0.001***	0.03 – 0.08	1.058
EDA	0.021	0.009	2.460	0.014*	0.004 – 0.04	1.021
IBI	−0.007	0.010	−0.673	0.501	−0.03 – 0.01	0.993
	*Disgust*					
	Coeff	SE	z	*p*	95% CI	Odds ratio
Swallowing	0.033	0.008	3.979	<0.001***	0.02 – 0.05	1.033
RSA	−0.014	0.008	-1.753	0.080	−0.03 – 0.002	0.986
Respiration	−0.006	0.009	-0.615	0.538	−0.02 – 0.01	0.994
EGG	−0.053	0.012	-4.454	<0.001***	−0.08 – −0.03	0.948
EDA	0.024	0.008	2.846	0.004**	0.008 – 0.04	1.024
IBI	0.002	0.010	0.164	0.870	−0.02 – 0.02	1.001
	*Neutral*					
	Coeff	SE	z	*p*	95% CI	Odds ratio
Swallowing	−0.041	0.012	−3.457	0.001***	−0.06 – −0.02	0.960
RSA	−0.020	0.008	−2.510	0.012*	−0.04 – −0.004	0.980
Respiration	−0.011	0.010	−1.132	0.257	−0.03 – 0.008	0.989
EGG	0.038	0.011	3.388	0.001***	0.02–0.06	1.039
EDA	0.016	0.009	1.926	0.054	−0.0002 – 0.03	1.017
IBI	−0.014	0.010	−1.427	0.154	−0.03 – 0.005	0.986
	*Pride*					
	Coeff	SE	z	*p*	95% CI	Odds ratio
Swallowing	0.002	0.009	0.233	0.816	−0.02 – 0.02	1.002
RSA	-0.021	0.008	−2.501	0.012*	−0.04 – −0.004	0.979
Respiration	0.012	0.009	1.290	0.197	−0.006 – 0.03	1.012
EGG	−0.075	0.012	−6.047	<0.001***	−0.10 – −0.05	0.928
EDA	0.030	0.009	3.435	0.001***	0.01 – 0.05	1.030
IBI	−0.027	0.010	−2.664	0.008**	−0.05 – −0.007	0.973
	*Sad*					
	Coeff	SE	Z	*p*	95% CI	Odds ratio
Swallowing	0.022	0.008	2.687	0.007**	0.006 – 0.04	1.022
RSA	−0.023	0.008	−2.840	0.005**	−0.04 – −0.007	0.978
Respiration	−0.008	0.009	−0.822	0.411	−0.03 – 0.01	0.992
EGG	−0.042	0.011	−3.656	<0.001***	−0.06 – −0.02	0.959
EDA	0.017	0.008	1.969	0.049*	0.0001– 0.03	1.017
IBI	−0.003	0.010	−0.295	0.768	−0.02 – 0.02	0.997

*Significant at the 0.05 level; **Significant at the 0.01 level; ***Significant at the 0.001 level.

**Table 3:** Median and standard deviation for POMP and absolute values for all psychophysiologic signals

	POMP Mdn	POMP SD	Absolute value Mdn	Absolute value SD
Electrogastrography				
Amusement	53.330	11.989	3.255	0.469
Disgust	31.990	11.799	2.650	0.422
Neutral	51.020	11.981	3.262	0.498
Pride	27.260	12.020	2.612	0.459
Sadness	35.600	10.221	2.725	0.451
Guilt	40.390	20.508	2.877	0.715
Electrodermal activity				
Amusement	37.825	17.758	2.005	2.843
Disgust	36.967	20.672	2.027	3.046
Neutral	31.600	17.008	2.125	2.748
Pride	38.599	22.242	2.288	2.849
Sadness	33.518	17.669	2.109	2.802
Guilt	26.762	19.023	1.758	2.916
Swallowing electromyography				
Amusement	9.375	10.400	0.222	0.345
Disgust	25.000	18.983	0.667	0.590
Neutral	7.143	9.333	0.143	0.323
Pride	12.500	19.573	0.333	0.633
Sadness	22.222	17.764	0.571	0.564
Guilt	0	23.602	0	0.626
Respiratory sinus arrhythmia				
Amusement	50.548	17.844	6.212	1.557
Disgust	51.740	19.232	6.225	1.440
Neutral	43.688	19.435	6.070	1.526
Pride	49.458	19.434	6.103	1462
Sadness	48.775	18.041	6.094	1.570
Guilt	56.755	21.967	6.339	1.433
Interbeat interval				
Amusement	53.624	14.752	857.241	114.316
Disgust	55.965	16.009	861.104	117.156
Neutral	54.473	14.220	867.112	116.350
Pride	50.325	16.844	848.030	117.440
Sadness	54.547	13.782	858.446	115.050
Guilt	53.824	18.569	865.990	113.655
Respiration rate				
Amusement	57.490	13.726	17.682	3.876
Disgust	49.903	16.818	17.061	4.108
Neutral	47.439	15.348	16.906	3.556
Pride	56.732	19.518	17.848	3.812
Sadness	47.307	15.631	16.868	3.606
Guilt	50.000	19.031	17.159	3.950

#### Electrogastrography

**Fig. 3:**
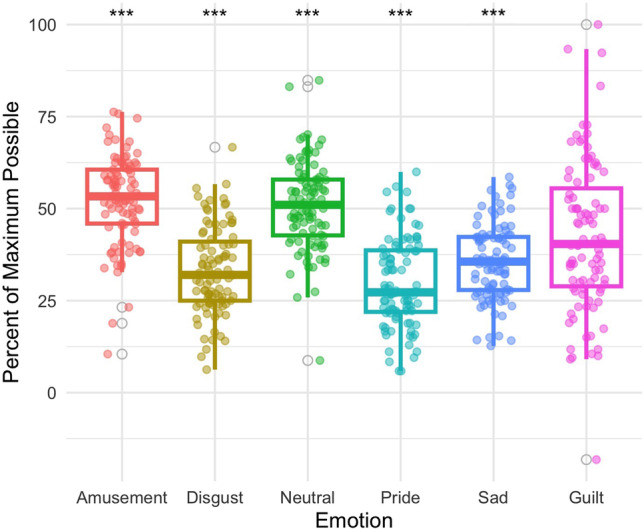
Electrogastrography was found to distinguish guilt (Mdn = 40.390) from amusement (Mdn = 53.330) *p* < 0.001, disgust (Mdn = 31.990 *p* < 0.001, neutral (Mdn = 51.020) *p* < 0.001, pride (Mdn = 27.260) *p* < 0.001, and sadness (Mdn = 10.221) *p* < 0.001. ***Significant at the 0.001 level

EGG contributed to the distinction between guilt and amusement (*z* = 4.814, *p* < 0.001), disgust (*z* = −4.454, *p* < 0.001), neutral (*z* = 3.388, *p* < 0.001), pride (*z* = −6.047, *p* < 0.001), and sadness (*z* = −3.656, *p* < 0.001), indicating that EGG is slower in guilt relative to amusement, pride, and neutral and faster relative to disgust and sadness (Fig. 3).

#### Electrodermal activity

**Fig 4:**
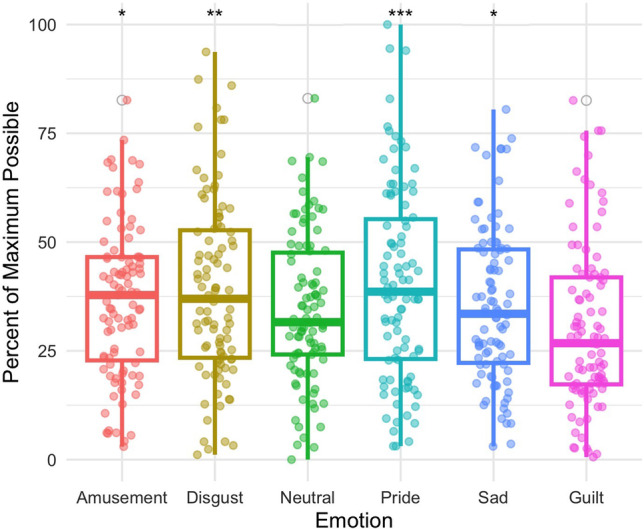
Electrodermal activity distinguished guilt (Mdn = 26.762) from amusement (Mdn = 37.825) *p* = 0.014, disgust (Mdn = 36.967) *p* = 0.004, and pride (Mdn = 38.599) *p* < 0.001. *Significant at the 0.05 level; **Significant at the 0.01 level; ***Significant at the 0.001 level

EDA contributed to the distinction between guilt and amusement (*z* = 2.460, *p* = 0.014), disgust (*z* = 2.846, *p* = 0.004), pride (*z* = 3.435, *p* < 0.001), and sadness (*z* = 1.969, *p* < 0.049), indicating that EDA magnitude is lower in guilt relative to those emotions. There was no significant effect detected for EDA comparing guilt to neutral (*z* = 1.924, *p* = 0.054; Fig. 4).

#### Swallowing electromyography

**Fig. 5:**
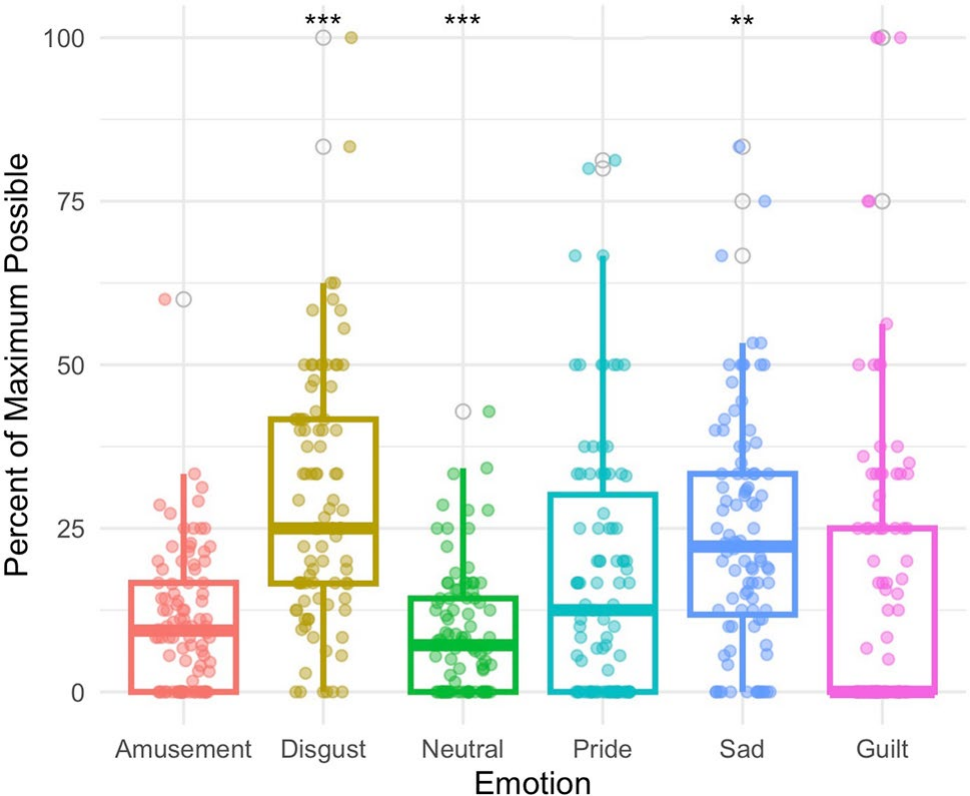
Swallowing rates distinguished guilt (Mdn = 0) from disgust (Mdn = 25) *p* < 0.001, neutral (Mdn = 7.143) *p* < 0.001, and sadness (Mdn = 22.222), *p* = 0.005. **Significant at the 0.01 level; ***Significant at the 0.001 level.

Swallowing rate contributed to the distinction between guilt and disgust (*z* = 3.979, *p* < 0.001), neutral (*z* = −3.457, *p* < 0.001), and sadness (*z* = 2.687, *p* = 0.007), indicating that in guilt swallowing is lower relative to disgust and sadness, and higher relative to neutrality. No significant effect was detected for swallowing rate comparing guilt to amusement (*z* = −1.817, *p* = 0.069) or to pride (*z* = 0.233, *p* = 0.816; Fig. 5).

#### Respiratory sinus arrhythmia

**Fig. 6:**
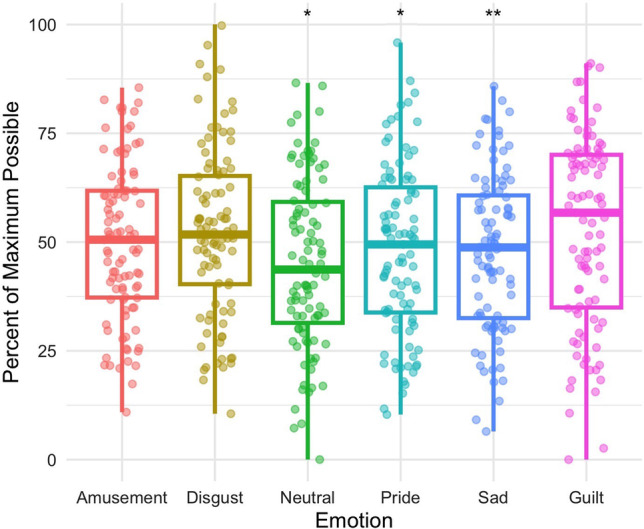
RSA showing a significant difference when comparing guilt (Mdn = 56.755 to sadness (Mdn = 48.775) *p* < 0.001, guilt to pride (Mdn = 49.458) *p* < 0.001, and guilt to neutral (Mdn = 43.688) *Z =* 2529, *p* = 0.007, *r* = 0.306. *Significant at the 0.05 level; **Significant at the 0.01 level

RSA contributed to the distinction between guilt and neutral (*z* = −2.510, *p* = 0.012), pride (*z* = −2.501, *p* = 0.012), and sadness (*z* = −2.840, *p* = 0.005), indicating greater RSA and thus greater PSNS heart control in guilt. There was no significant effect found comparing guilt to amusement (*z* = −0.552, *p* = 0.581) or disgust (*z* = −1.753, *p* = 0.080; Fig. 6).

#### Interbeat interval

IBI contributed to the distinction between guilt and pride (*z* = −2.664, *p* = 0.008) indicating greater IBI and slowing of heart rate in guilt relative to pride. There was no significant effect detected for IBI when comparing guilt to amusement (*z* = −0.673, *p* = 0.501), disgust (*z* = 0.164, *p* = 0.870), neutral (*z* = −1.427, *p* = 0.154) or sadness (*z* = −0.295, *p* = 0.768).

#### Respiration rate

Respiration rate contributed to the distinction between guilt and amusement (*z* = 3.605, *p* < 0.001), indicating that respiration is lower in guilt relative to amusement. There was no effect detected for respiration comparing guilt to disgust (*z* = −0.615, *p* = 0.538), neutral (*z* = −1.132, *p* = 0.257), pride (*z* = 1.290, *p* = 0.197), or sadness (*z* = −0.822, *p* = 0.411).

## General discussion

That the ANS is responsive to basic emotional states and produces patterns of activation that are both identifiable and observable has been well established (Christie & Friedman, 2004; Kreibig, 2010; Levenson, 2014). We sought to identify whether guilt also has an autonomic signature distinguishable from those of other emotions and to delineate the specific pattern of autonomic activations that accompany the experience of guilt. This study has provided perhaps the first evidence in healthy adults that guilt, too, is associated with an autonomic response that is detectable and unique. First, the results establish that the cognitive experience of guilt occurs alongside a detectable pattern of autonomic outflow. Second, we found that this pattern of autonomic outflow was different from the patterns produced by the comparison emotions.

Overall, the pattern of autonomic responding observed in guilt indicated a mixed picture of sympathetic and parasympathetic activation. This was observed in comparisons of guilt to positive (amusement, pride), negative (disgust, sadness), and neutral conditions. Relative to guilt, each emotional comparison condition had at least one psychophysiological signal for which the SNS activation or PSNS withdrawal predominated and at least one in which PSNS activation or SNS withdrawal predominated. This finding was unexpected, as we hypothesized that guilt would be a highly autonomically activating emotion that would tend towards SNS activation and PSNS withdrawal. We put forth candidate explanations for this pattern of autonomic activity based on the four psychophysiological signals that contributed most strongly to the pattern of activation observed to distinguish guilt from the comparison emotions. These were electrogastrogram (EGG), swallowing rate, respiratory sinus arrhythmia (RSA), and electrodermal activity (EDA).

The EGG was the only signal where guilt differed from all comparison emotions. Relative to guilt, EGG activity was found to be greater, i.e., the regular contractile rhythm of the stomach was more frequent, in amusement, pride, and neutrality, suggesting heightened activity of the SNS and decreased activity of the PSNS during guilt relative to those emotions and a neutral state (Stern, 2002). By contrast, EGG activity was relatively lower in disgust and sadness, suggesting greater relative influence of the PSNS and relatively lesser SNS activity during guilt (Stern, 2002). This pattern distinguished guilt clearly from both the neutral and positive conditions and aligns it more closely to the negative conditions. Guilt, much like disgust, thus appears to be associated with reduced amplitude of the EGG signal, although this effect may occur less strongly in guilt than disgust (Shenhav & Mendes, 2014; Vianna & Tranel, 2006). This may suggest a tendency to bradygastria in negative emotions beyond disgust.

Swallowing rate was found to be lower in guilt relative to disgust or sadness, and higher in guilt relative to neutral. A decrease in swallowing in guilt relative to disgust and sadness suggests relatively greater SNS activation in guilt and could be related to the subjective dry mouth, as well as actual decrease in salivation, that has been reported during emotional upset, anxiety, and guilt (Bates & Adams, 1968; Gemba et al., 1996; Gholami et al., 2017; Kubany et al., 1996). By contrast, sadness often involves swallowing down tears, mucous, or the lump in the throat produced by crying or the urge to cry (Hepburn, 2004; Mori & Iwanaga, 2017; Vingerhoets et al., 1997). Similarly, when disgusted one must swallow excess saliva produced by nausea or the urge to vomit (Horn, 2008; Hornby, 2001; van Overveld et al., 2009). Dry mouth, combined with swallowing triggers in the other emotions, may explain this observed difference in swallowing response. This finding provides a potential marker of guilt’s autonomic distinctness from sadness and disgust. Both disgust and sadness have, at times, been conceptualized as progenitors for, or the predominant emotion underlying, guilt (Power & Dalgleish, 2015; Turner, 2007). As negative, aversive emotions that can be elicited in moral situations and stirred by the suffering of others, sadness (for others, for oneself, for the situation) and disgust (moral disgust, disgust with oneself) often co-occur with guilt (Malti et al., 2016; Olatunji et al., 2012; Ottaviani et al., 2018). Interpersonally, disgust might be elicited by the moral transgressions of others (e.g., cheating or sexual deviance) that could elicit guilt in oneself, suggestive of a close relationship between the two emotions (Bomyea & Allard, 2017; Chapman & Anderson, 2013; Olatunji & Sawchuk, 2005). Similarly, sadness often is elicited by witnessing another being transgressed against, and the sadness of others can stir guilt if the observer attributes blame for that sadness to themselves (Roos et al., 2011; Turner, 2007; Turner & Stets, 2006).

RSA differed between guilt and neutral, pride, and sadness. The greater RSA in guilt relative to these emotions suggests a vagally-mediated deceleration of the heart and thus suggests PSNS involvement in guilt (Butler et al., 2006). This aligns well with existing research, which has associated heart rate deceleration with moral emotions and suggests that it is observed particularly in emotions that are both negative and moral (Colasante et al., 2018; Malti et al., 2016). Previous research has suggested that greater vagal tone at rest and in response to emotional stimuli is correlated with attention, arousal, and emotional regulation (Balzarotti et al., 2017; Butler et al., 2006; Frazier et al., 2004; Park & Thayer, 2014). Greater RSA in guilt relative to the comparison emotions may reflect increased attention to negative self-information or use of emotional regulation strategies to respond to negative feelings (Sharvit et al., 2015; van Dijk et al., 2017).

EDA magnitude was higher in amusement, disgust, pride, and sadness relative to guilt. This finding was unexpected, because guilt is typically conceptualized as an arousing and motivating experience that would be expected to increase EDA reactivity in healthy adults (Boucsein, 2012; Bradley et al., 2001; Cuthbert et al., 2000). Instead, these results suggest that there is withdrawal of SNS activation of the skin in guilt relative to these other emotions. The reason for this result is unclear, although a previous study found a nonsignificant decline in EDA signal in children between the anticipation and act of transgression (Colasante et al., 2018), whereas another suggests that EDA is less reactive to negative social stimuli compared with negative nonsocial stimuli or positive social stimuli (Britton et al., 2006). It may be, therefore, that this finding is a reflection of the negative, social nature of guilt. There also is evidence that emotions that evoke passivity, or to which no immediate action is available, such as depression or contentment, tend to show SNS withdrawal and PSNS activation (Kreibig, 2010). It is possible that, during the task, participants became passive, because they knew that no action could be taken to alleviate guilt in the short-term, and they had no way to halt the guilt-inducing behaviour. Thus, SNS withdrawal predominated as there was no opportunity and therefore no need for the ANS to prepare a behavioural response. Further research is needed to confirm and develop upon these findings.

The other psychophysiological signals distinguished between guilt and only one other emotion. IBI distinguished between guilt and pride; in guilt IBI was lengthened relative to pride, suggestive of a comparative deceleration in heart rate, and therefore an increase in PSNS control of the heart, in guilt. A similar lengthening of IBI has been observed in sadness relative to happiness and in fear relative to neutrality; it appears to be related to emotional valence rather than arousal (Frazier et al., 2004; Fredrickson & Levenson, 1998; Krumhansl, 1997). Because guilt and pride are both social emotions that differ in terms of valence, IBI might be useful to distinguish along the spectrum of negativity and positivity in social emotions. Respiration rate was lower in guilt compared with amusement, suggesting an increase in relative PSNS control of breathing in guilt. This difference might be due to laughter, which participants often engaged in during amusement videos but not during guilt videos. However, there was no effect of crying on respiration during sadness videos, whereas participants typically did not cry during guilt videos.

Overall, the observed pattern of EGG, swallowing rate, RSA, and EDA in guilt relative to the comparison emotions suggests a mixture of SNS and PSNS activation and withdrawal across the various effectors measured, with relative activations of either branch dependent on the comparison emotion. This aligns with autonomic patterns observed for basic emotions, such as sadness, which displays a mix of SNS and PSNS activity, and even coactivation (Gross et al., 1994; Hendriks et al., 2007; Mori & Iwanaga, 2017).

## Limitations

One potential limitation of this study is the semantic ambiguity around the word “guilt.” Often, “guilt” and “shame” are used interchangeably in casual speech, despite differences that have been well characterized in the literature (Tangney, 1992; Tangney et al., 1996). Potential conflation of terms also occurs to a lesser extent between guilt and embarrassment (Tangney et al., 1996; Withers & Sherblom, 2008). These social emotions also often co-occur, resulting in challenges in distinguishing between them in naturalistic settings (Smith & Ellsworth, 1985). In the present study, participants were able to choose guilt, shame, or embarrassment as their main or secondary emotions, and trials identified as eliciting primarily shame or embarrassment were removed from analysis as not-guilt. Despite these measures, it is possible that contamination between these terms occurred. A future study might instruct participants on the precise definitions of key emotional terms to ensure that participants are generating the clearest labels for each emotional experience. While the study presents perhaps the first comprehensive evaluation of ANS patterns during a negative social emotion, in the absence of other negative social emotion comparison conditions, we do not draw conclusions about guilt’s uniqueness within that category. Building on the findings, future studies, including a comparison condition with shame, embarrassment, or both, may next delineate whether other negative social emotions share the unique ANS pattern observed during guilt. Another potential limitation is that measurements of emotional intensity were not taken after each video. Instead, participants provided an estimate of the average overall strength of the emotions experienced at the end of the video task. It is therefore not possible to know if the psychophysiological patterns detected were parametrically correlated with the intensity of emotion experienced. Finally, the psychophysiological scores were taken as arithmetic means of the physiological response for the identified window of emotion during each video. This represents a conservative estimate of the psychophysiological response. Individuals might not consistently experience the target emotion, might experience fluctuating levels of emotional intensity or might experience different primary emotions throughout the video (Davydov et al., 2011). Future studies could examine more granular epochs to attempt to capture specific psychophysiological responding that might be lost to averaging. Finally, the specific combined patterns of the psychophyisologic emotions are of particular interest and relevance to the question of whether a distinct profile exists for guilt. Towards this goal, a MANOVA was used as the initial omnibus analytic approach, because it would capture multivariate differences in the psychophysiologic signals associated with each emotion. Because the MANOVA did indicate significant differences amongst the emotions, ANOVAs were used to delineate the direction of each physiologic signal to evaluate its contribution to the multivariate differences. While we did not have an independent sample to establish a classification model, we consider this the next important step to validate the pattern of findings for the signals in the present study and establish their predictive power in distinguishing between emotions.

## Conclusions

This study sought to address the current gap in the literature around the physiological nature of guilt by investigating which autonomic signals are associated with guilt relative to other emotions in healthy adults. Guilt was accompanied by a pattern of SNS and PSNS activations, particularly EGG, swallowing rate, and EDA. These findings lay the groundwork for development and validation of classification models, and future studies exploring disorders with excesses or paucities of guilt, as in obsessive-compulsive disorder, posttraumatic stress disorder, psychopathy, or frontotemporal dementia. Additional future directions include investigating the autonomic responses identified in this study in children who are developing guilt, or in populations outside of North America, who might have different conceptualizations and experiences of guilt.

## Supplementary Information

ESM 1 (DOCX 50 kb)
