# Supplementary material for: The psychophysiology of guilt in healthy adults
Source: Cogn Affect Behav Neurosci. 2023 Mar 25;23(4):1192–209. doi: 10.3758/s13415-023-01079-3 (PMC10400478; doi:10.3758/s13415-023-01079-3)
Supplement: Supplementary file 1 — (DOCX 50 kb) [file 13415_2023_1079_MOESM1_ESM.docx]

**Supplemental Material**

### **Sample questions and response options for opinions and behaviour questionnaire**

Do you think that it is important to help the poor or underprivileged?

1. Yes
2. No
3. Don’t know

In the past 12 months have you engaged in formal or informal unpaid activities aimed at conservation or preservation of the environment or wildlife?

1. Yes
   1. How much time on average per week, in hours? _____
2. No
3. Don’t know

In the past 12 months, have you sped while driving, passed without adequate space, or otherwise driven unsafely?

1. Yes, frequently
2. Yes, on a few occasions
3. Yes, rarely
4. No

On a scale from 1 to 5, how important is finding a cure for cancer to you?

| 1 | 2 | 3 | 4 | 5 |
| --- | --- | --- | --- | --- |
| Not important at all |  |  |  | Very important |

Do you think that having new things is important?

1. Yes
2. No

On a scale from 1 to 5, how important is it to you to carry on Canadian traditions, customs, and holidays?

| 1 | 2 | 3 | 4 | 5 |
| --- | --- | --- | --- | --- |
| Not important at all |  |  |  | Very important |

Do you usually use a dryer, or do you air dry clothing where possible?

1. Usually use a dryer in both summer and winter
2. Usually use a dryer in winter, but not in summer
3. Do not have/do not use a dryer

###

### **Supplemental Table 1.** Video clip details

| Video Name | Target Emotion | Length | Analyzed Length* | Topic | Source Agency/Film | Source Country |
| --- | --- | --- | --- | --- | --- | --- |
| 60 Seconds Blood Donation | Guilt | 60s | 45s | Blood Donation | Canadian Blood Services | Canada |
| Africare - Shoeboxes | Guilt | 30s | 20s | Poverty/3rd World | Africare | USA |
| Black Friday | Guilt | 30s | 8s | Poverty/Canada | Salvation Army | Canada |
| WWF Laptop | Guilt | 2m15s | 1m20s | Environment/Climate Change | World Wildlife Fund | USA |
| WWF T-Shirt | Guilt | 1m50s | 1m30s | Environment/Climate Change | World Wildlife Fund | USA |
| Don't Almost Give-Jack | Guilt | 30s | 20s | Poverty/Canada | Ad Council | USA |
| Let Them Figure It Out | Guilt | 60s | 53s | Environment/Climate Change | Government of Ontario | Canada |
| One Child | Guilt | 1m40s | 1m20s | Poverty/3rd World | Save the Children | UK |
| Strawberry Wasted | Guilt | 60s | 15s | Food Waste | Ad Council | USA |
| Without... | Guilt | 1m15s | 57s | Poverty/3rd World | Save the Children | USA |
| Go First | Amusement | 30s | 18s | Advertisement | Cars.com | USA |
| Dad Makes Cookies | Amusement | 30s | 21s | Adoption | Ad Council | USA |
| Charlie Sheen | Amusement | 30s | 20s | Advertisement | DirecTV | USA |
| French | Amusement | 45s | 36s | Adoption | Ad Council | USA |
| Haircut | Amusement | 30s | 20s | Adoption | Ad Council | USA |
| Sinking | Amusement | 40s | 30s | Advertisement | Berlitz | USA |
| Drinking 6000 Blended Maggots | Disgust | 45s | 40s | Non-food consumption | Where's My Challenge | UK |
| Custard | Disgust | 60s | 45s | Food contamination | Braindead | New Zealand |
| The Poop Lady | Disgust | 55s | 47s | Food contamination | Hoarders | USA |
| Rotting Chicken | Disgust | 1m16s | 1m12s | Rotting food | Home video/ David0101010 | Unknown |
| Rotting Meat | Disgust | 1m36s | 1m31s | Rotting food | Home video/ agnozja | Unknown |
| Toxic Food Environment | Disgust | 20s | 17s | Vomiting | Supersize Me | USA |
| Cotton | Neutral | 1m | 30s | Informational | How It's Made | Canada |
| Erasers | Neutral | 45s | 23s | Informational | How It's Made | Canada |
| Fibre Optics | Neutral | 40s | 20s | Informational | How It's Made | Canada |
| Playing Cards | Neutral | 35s | 18s | Informational | How It's Made | Canada |
| Rubber Gloves | Neutral | 40s | 20s | Informational | How It's Made | Canada |
| Stickers | Neutral | 35s | 18s | Informational | How It's Made | Canada |
| Fly the Flag | Pride | 60s | 52s | Canadian identity | Air Canada | Canada |
| Greatest Human Achievements | Pride | 1m55s | 1m30s | World unity/human excellence | Questar Video | UK |
| I Am Canadian | Pride | 1m | 39s | Canadian identity | Molson Canadian | Canada |
| Made From Canada | Pride | 45s | 37s | Canadian identity | Molson Canadian | Canada |
| Peacekeeping | Pride | 1m10s | 53s | Canadian Peacekeeping | Royal Canadian Mounted Police | Canada |
| The Anthem | Pride | 1m30s | 1m18s | World unity | Samsung Mobile | USA |
| 12 Days of Christmas | Sadness | 1m30s | 1m9s | Anti-drunk driving | Transport Accident Commission | Australia |
| Dying Wife | Sadness | 1m20s | 1m15s | Death of a loved one | Home video/ Erin Solari | USA |
| Madly in love | Sadness | 1m45s | 1m10s | Loss of a loved one to dementia | Human Kind | USA |
| Last Minutes with Oden | Sadness | 1m10s | 59s | Death of a pet | Phos Pictures | USA |
| Notification | Sadness | 45s | 25s | Death of a loved one | The Messenger | USA |
| SickKids vs Cancer | Sadness | 40s | 19s | Childhood cancer | SickKids | Canada |

*Analyzed length indicates the time window of analysis which began at average onset of emotion experienced based on piloting, and ended at the conclusion of the video clip. For example, for a 60s second video clip with an analyzed length of 45s, the time window analyzed began 15 seconds after the start of the clip, and continued until the video ended.

### **Supplemental Table 2.** Feedback Statements

| Video | Statement |
| --- | --- |
| *Black Friday* | You like to have new things |
| *One Child* | You donate less than the average Canadian to international relief efforts |
| *Don't Almost Give- Jack* | You could do more to help people in Canada |
| *Africare-Shoeboxes* | You don’t want to be repeatedly asked to donate money |
| *Without...* | You sometimes ignore charity appeals |
| *Strawberry Wasted* | You waste much more food than average |
| *WWF Laptop* | You waste more energy than average |
| *WWF T-Shirt* | Your laundry habits waste more water than two thirds of respondents |
| *60 Seconds Blood Donation* | You think blood and organ donation is as important as most Canadians |
| *French* | You find humourous ads more memorable than the average Canadian |
| *Sinking* | You can always learn a new language |
| *Charlie Sheen* | You are as influenced by celebrity endorsement as most Canadians |
| *Dad Makes Cookies* | You bake less than the average Canadian |
| *Go First* | You are as influenced by ads as most people |
| *Haircut* | Your opinion of adoption is shared by half of Canadians |
| *Rotting Chicken* | You worry about rotted food as much as the average person |
| *Drinking 6000 Blended Maggots* | You feel as sick about maggots as the average person |
| *Rotting Meat* | You would never eat rotted meat |
| *The Poop Lady* | You should not eat contaminated food |
| *Toxic Food Environment* | You are as anxious about vomiting as the average person |
| *Custard* | You would not eat food contaminated with bodily fluids |
| *Cotton* | You will see a video about cotton thread |
| *Rubber Gloves* | You will see a video about rubber gloves |
| *Fibre Optics* | You will see a video about fibre optics |
| *Erasers* | You will see a video about erasers |
| *Playing Cards* | You will see a video about playing cards |
| *Stickers* | You will see a video about stickets |
| *I Am Canadian* | You are in Canada |
| *Made From Canada* | Your sense of Canadian identity is as strong as average |
| *Anthem* | Your sense of connection to the world is stronger than average |
| *Peacekeeping* | You think peacekeeping is as important as most Canadians |
| *Fly the Flag* | You feel connected to Canada |
| *Greatest Human Achievements* | You are proud of humanity’s achievements |
| *Dying Wife* | You get sad about as easily as other people |
| *SickKids vs Cancer* | You think fighting cancer is important |
| *Last Minutes with Oden* | You feel sorry for people who have recently lost a pet |
| *12 Days of Christmas* | You should not drive unsafely |
| *Notification* | You feel sorry for people who have recently lost a child |
| *Madly in love* | You feel sad when someone else is sad about as often as other people |

**Supplemental Table 3.** Means, standard deviations, and ranges for trait and state characteristics. 108 participants (55 female) completed the Guilt Inventory and EQ; 72 participants (27 female) completed the STAI and BPQ.

|  | M | SD | Range |
| --- | --- | --- | --- |
| Guilt Inventory | 128.565 | 21.377 | 75-190 |
| Empathy Quotient | 43.870 | 10.588 | 17-73 |
| State-Trait Anxiety Inventory  *State* | 28.417 | 7.881 | 20-55 |
| *Trait* | 38.972 | 11.835 | 20-74 |
| Body Perception Questionnaire  *Body Awareness* | 71.694 | 21.677 | 30-118 |
| *Supradiaphragmatic reactivity* | 22.347 | 7.710 | 15-48 |
| *Subdiaphragmatic reactivity* | 9.431 | 3.297 | 6-19 |

**Supplemental Table 4.** Total counts of primary emotions identified by intended emotion

| Intended Emotion  Endorsed Emotion | Amusement | Disgust | Guilt | Neutral | Pride | Sadness |
| --- | --- | --- | --- | --- | --- | --- |
| Amusement | 372 | 46 | 12 | 87 | 56 | 2 |
| Anger | 2 | 2 | 45 | 1 | 3 | 11 |
| Contempt | 6 | 5 | 25 | 6 | 6 | 4 |
| Disgust | 1 | 445 | 17 | 1 | 0 | 1 |
| Embarrassment | 3 | 7 | 45 | 0 | 2 | 0 |
| Fear | 3 | 4 | 27 | 1 | 1 | 7 |
| Guilt | 2 | 1 | 222 | 0 | 1 | 9 |
| Happiness | 65 | 1 | 14 | 27 | 87 | 26 |
| Neutral | 103 | 44 | 147 | 435 | 92 | 30 |
| Pride | 5 | 0 | 22 | 6 | 314 | 2 |
| Sadness | 2 | 8 | 266 | 0 | 2 | 470 |
| Shame | 0 | 1 | 80 | 0 | 0 | 2 |

**Supplemental Table 5.** Total numbers of primary emotions endorsed and accuracy to the video’s intended emotion, with total emotions broken down by valence and social nature

|  | Amusement | Disgust | Guilt | Neutral | Pride | Sadness |
| --- | --- | --- | --- | --- | --- | --- |
| Total Emotion Endorsements | 564 | 564 | 922 | 564 | 564 | 564 |
| Total Non-target Emotions | 192 | 119 | 700 | 129 | 250 | 94 |
| Average Target Accuracy | 65.957% | 78.901% | 24.078% | 77.128% | 55.674% | 83.333% |
| Total Positive Emotions | 442 | 47 | 48 | 120 | 401 | 30 |
| Total Negative Emotions | 19 | 473 | 727 | 9 | 15 | 504 |
| Total Negative Social Emotions | 5 | 9 | 347 | 0 | 3 | 11 |
| Total Positive Social Emotions | 5 | 0 | 22 | 6 | 314 | 2 |

**Supplemental Table 6.** Counts of secondary emotions identified by intended emotion

| Intended Emotion  Endorsed Emotion | Amusement | Disgust | Guilt | Neutral | Pride | Sadness |
| --- | --- | --- | --- | --- | --- | --- |
| Amusement | 127 | 67 | 35 | 84 | 112 | 13 |
| Anger | 9 | 66 | 125 | 3 | 2 | 69 |
| Contempt | 11 | 73 | 65 | 12 | 22 | 18 |
| Disgust | 3 | 149 | 56 | 3 | 1 | 11 |
| Embarrassment | 19 | 41 | 128 | 1 | 7 | 13 |
| Fear | 12 | 62 | 70 | 3 | 7 | 90 |
| Guilt | 12 | 13 | 306 | 5 | 3 | 59 |
| Happiness | 177 | 8 | 36 | 43 | 301 | 77 |
| Neutral | 132 | 54 | 97 | 182 | 70 | 57 |
| Pride | 33 | 0 | 25 | 16 | 185 | 25 |
| Sad | 13 | 45 | 229 | 3 | 13 | 135 |
| Shame | 8 | 28 | 281 | 1 | 2 | 29 |

**Supplemental Table 7.** Total numbers of secondary emotions endorsed, with total emotions broken down by valence and social nature

|  | Amusement | Disgust | Guilt | Neutral | Pride | Sadness |
| --- | --- | --- | --- | --- | --- | --- |
| Total Secondary Emotions Endorsed | 556 | 606 | 1453 | 356 | 725 | 596 |
| Total Non-target Emotions | 429 | 457 | 1147 | 174 | 540 | 461 |
| Total Positive Emotions | 337 | 75 | 96 | 143 | 598 | 115 |
| Total Negative Emotions | 87 | 477 | 1260 | 31 | 57 | 424 |
| Total Negative Social Emotions | 39 | 82 | 715 | 7 | 12 | 101 |
| Total Positive Social Emotions | 33 | 0 | 25 | 16 | 185 | 25 |
